# Nanoemulsion as a Platform for Iontophoretic Delivery of Lipophilic Drugs in Skin Tumors

**DOI:** 10.3390/pharmaceutics10040214

**Published:** 2018-11-04

**Authors:** Luciana Facco Dalmolin, Renata F. V. Lopez

**Affiliations:** School of Pharmaceutical Sciences of Ribeirão Preto, University of Sao Paulo, Av. Cafe s/n, Ribeirao Preto 14040-903, SP, Brazil; lucianafd@usp.br

**Keywords:** nanoemulsion, lipophilic drugs, iontophoresis, zinc phthalocyanine, skin penetration

## Abstract

Lipophilic drugs do not usually benefit from iontophoresis mainly because they do not solubilize in aqueous formulations suitable for the application of electric current. To explore the influence of iontophoresis on penetration of these drugs, a cationic nanoemulsion was developed to solubilize zinc phthalocyanine (ZnPc), a promising drug for the treatment of skin cancer. To verify the influence of particle size on iontophoresis, an emulsion of nanoemulsion-like composition was also developed. The formulations were characterized and cutaneous and tumor penetration studies were performed in vitro and in vivo, respectively. With particles of about 200 nm, the nanoemulsion solubilized 2.5-fold more ZnPc than the 13-µm emulsion. At the same concentration of ZnPc, in vitro passive penetration studies showed that the nanoemulsion increased, after 1 h of treatment, by almost 4 times the penetration of ZnPc into the viable layers of the skin when compared to the emulsion, whereas iontophoresis of nanoemulsion resulted in a 16-fold increase in ZnPc penetration in only 30 min. An in vivo study in a murine model of melanoma showed that ZnPc reached the tumor after iontophoresis of the nanoemulsion. Therefore, iontophoresis of nanoemulsions appears to be a promising strategy for the topical treatment of tumors with lipophilic drugs.

## 1. Introduction

Skin cancer is one of the most diagnosed types of cancer in the world. Among the therapies currently available for its treatment, topical photodynamic therapy (PDT) stands out for its applicability and efficiency as it allows the delivery of the drug directly to the tumor site, thereby avoiding systemic adverse effects in addition to being a non-invasive therapy [1,2].

Topical PDT involves the application of a photosensitizing agent (PS) to the skin that covers the tumor. The PS must penetrate the skin and accumulate in the tumor cells. Once there, the tumor needs to be irradiated with a light at a wavelength suitable to excite the PS, thus generating reactive oxygen species (ROS) and singlet oxygen. These lead to indirect toxic effects, such as damage to tumor vasculature [3,4] and induction of immune response [5] and direct effects, such as necrosis and apoptosis of tumor cells [6,7,8].

Zinc Phthalocyanine (ZnPc) is a PS that absorbs light in a wavelength range between 600 and 800 nm, in which the body’s endogenous chromophores, such as melanin, produce low interference and allow this light to reach the deeper skin layers, where skin tumors are located. With a high molar absorptivity coefficient (ε_670_ ≈ 10^5^ M^−1^ cm^−1^), the photoactive ZnPc generates numerous ROS and singlet oxygen (quantum yield of 0.67 in dimethyl sulfoxide (DMSO)), which are associated with subsequent cell death through the oxidation and degradation of cell components [9,10]. Moreover, the presence of zinc metal as the central ion extends the triplet lifetime of the PS, thus increasing the reactivity with the substrate [11]. Stable and non-toxic in the absence of light, the hydrophobic characteristic of ZnPc facilitates its cellular uptake and its accumulation in the mitochondria and lysosomes, a condition required for an efficient photodynamic effect [8,12,13,14].

However, the high lipophilicity of ZnPc impairs its systemic or topical administration. When applied to the skin, the high oil/water partition coefficient of ZnPc (logK > 8) results in its accumulation in the stratum corneum, preventing its diffusion and partition to the tumor cells [15,16,17].

Beyond the barrier of the stratum corneum, the tumor environment also provides a powerful barrier for drug transport [18]. Therefore, the application of a physical method such as iontophoresis may allow the transposition of these barriers for adequate drug skin penetration, creating alternative routes for the PS to reach the tumor cells.

Indeed, iontophoresis, i.e., the application of a weak but constant electric current, usually less than or equal to 0.5 mA/cm^2^ [19], to the skin has already been studied as a manner of increasing the skin transport of 5-aminolevulinic acid (5-ALA) [20,21], a precursor of an endogenous porphyrin, hydrophilic anionic and cationic porphyrins [22,23], and phthalocyanine tetrasulfonic acid [24,25], all of them being PSs used in PDT. However, these drugs are all hydrophilic, present smaller molar absorptivity coefficients than ZnPc, and consequently a less photodynamic efficiency.

Iontophoresis of ZnPc could therefore be promising to provide a higher concentration of this potent PS to tumor cells. Obviously, however, electric current is not conducted in nonpolar media, which are those capable of solubilizing high concentrations of ZnPc to be administered. Such non-polar media, such as mineral oil for example, also make difficult the PS partition from the formulation to the skin. Therefore, to take advantage of iontophoresis, ZnPc would need to be incorporated into a pharmaceutical dosage form that would allow the passage of electric current.

Oil-in-water emulsions (creams) are conventional pharmaceutical dosage forms widely used to deliver hydro or lipophilic drugs to the skin. As these emulsions have an aqueous outer phase, they allow the application of iontophoresis, while ZnPc can be incorporated into the internal oily phase. Similar drug delivery systems, but with a nano-sized inner phase, such as nanoemulsions, could also be used to carry ZnPc and be submitted to iontophoresis. These nano-sized emulsions provide a higher solubilization capacity than simple micellar dispersions and greater kinetic stability than coarse emulsions [26,27], making them a promising iontophoretic delivery platform for lipophilic drugs.

Therefore, this work aims to investigate the influence of iontophoresis on cutaneous and tumor penetration of ZnPc as a function of the characteristics of the ZnPc delivery system, being the delivery system a coarse emulsion or a nanoemulsion.

## 2. Materials and Methods

### 2.1. Chemicals

ZnPc, Poloxamer 188, and Fluoromount^®^ were purchased from Sigma-Aldrich (St Louis, MO, USA); dimethyl sulfoxide (DMSO) from Vetec (Rio de Janeiro, Brazil); Medium Chain Triglyceride (MCT), *N*-1,2-dioleoyloxy-3-trimethylammonium propane chloride (DOTAP), and egg phospholipids with 80% phosphatidylcholine (Lipoid E80) were purchased from Lipoid (Ludwigshafen, Germany); polysorbate 80, sodium chloride, potassium phosphate monobasic, and sodium phosphate dibasic from Synth (São Paulo, Brazil); and Tissue Tek (O.C.T. Compound) from Sakura (Torrance, CA, USA). The water used in all preparations was of Milli-Q grade with conductivity of 0.055 µS/cm (Millipore, France). 

### 2.2. Nanoemulsion and Emulsion Preparation

#### 2.2.1. Formulations for ZnPc Solubility Determination

The emulsion and nanoemulsion were prepared with the same composition. The oily phase was composed of 15% MCT, 1% Lipoid E80, 0.2% DOTAP, and an excess of ZnPc (10 mg). The aqueous phase contained 5% polysorbate 80 and 0.1% poloxamer 188, dispersed in water in a sufficient quantity to prepare 20 mL of the formulations. 

To prepare the coarse emulsion, the aqueous phase was simply added to the oily phase, both at 70 °C, under mechanical stirring (IKA Works Inc., Wilmington, NC, USA) for 5 min. 

To prepare the nanoemulsion, the coarse emulsion prepared as above was immediately sonicated for 5 min using a low frequency ultrasound (20 kHz, VCX 500; Sonics Materials Inc., Newtown, CT, USA) at 50% amplitude and pulse length of 1 s on, 1 s off. 

After 24 h the formulations were centrifuged (Thermo Scientific, Osterode, Germany) at 4000× *g* for 30 min to separate the unsolubilized ZnPc.

Blank nanoemulsion was also prepared in the same way but without ZnPc.

#### 2.2.2. Formulations for In Vitro Penetration Studies

Nanoemulsion and emulsion were prepared with the same concentration of ZnPc for in vitro experiments. Furthermore, to guarantee the transport of the electric current in the iontophoretic experiments, isotonic phosphate buffer solution (PBS) was used instead of water. ZnPc was dissolved in MCT at the concentration of about 30 μg/mL. The oily phase was composed of 15% MCT solution containing ZnPc, 1% Lipoid E80, and 0.2% DOTAP. The aqueous phase contained 5% polysorbate 80 and 0.1% poloxamer 188, dispersed in the isotonic PBS (pH 7.4, 10 mM, 0.9% NaCl) in sufficient quantity to prepare 20 mL of the formulations. The ZnPc coarse emulsion and nanoemulsion were prepared as described in Section 2.2.1.

### 2.3. Formulations Characterization

#### 2.3.1. Size and Polydispersity Index

For the nanoemulsion, the mean size, size distribution and polydispersity index (PDI) were determined by dynamic light scattering (DLS) (Malvern Zetasizer Nano ZS, Malvern, UK) at 25 °C after appropriate dilution (100-fold) in ultrapure water. The angle of backscattering was set at 90°. The refractive index employed for dispersant was 1.33. Medium viscosity was 0.8872 cP. For data recording, disposable cuvettes were used. 

For the emulsion, the mean size of the droplets and size distribution were determined by laser light diffraction (LS 13320 Laser Diffraction Particle Size Analizer, Beckman Coulter, CA USA). Polydispersity was analyzed based on the SPAN value, which was calculated according to Equation (1):

SPAN = (D90 − D10)/D50,(1)

where D10, D50 and D90 are the diameters at 10, 50 and 90% of cumulative volumes, respectively. The measurements were performed in triplicate using at least three different formulations.

#### 2.3.2. Zeta Potential

Zeta potential was determined by electrophoretic mobility under an electric field (Malvern Zetasizer Nano ZS, Malvern, UK) at 25 °C after appropriate dilution (100-fold) in ultrapure water. Measurements were made in the electrophoretic cell and a potential of ±150 mV was applied. Data analyses were performed using the Helmholtz–Smoluchowski approximation. The measurements were performed in triplicate.

#### 2.3.3. pH

The pH values were measured directly in the emulsions and nanoemulsions without dilution, using a digital pH meter (Digimed, São Paulo, Brazil).

#### 2.3.4. Morphology Analysis

The morphology analysis of the ZnPc nanoemulsion was performed with a transmission electron microscope (TEM) (JEOL JEM-100CX2, Tokyo, Japan). The sample preparation (without previous dilution) was negative staining using uranyl acetate solution 2%. The grids were dried for 24 h at a temperature of 25 °C and them observed with the TEM at 80 kV with magnifications of 20,000× and 100,000×.

#### 2.3.5. Stability Study

Physicochemical stability of ZnPc nanoemulsions was evaluated for 30 days at 4 °C. The parameters evaluated were mean diameter, PDI, zeta potential and pH. The mean values of these parameters were compared with those obtained at time zero. Data were subjected to statistical analysis employing one-way analysis of variance (ANOVA), followed by the Tukey test, and *p*-values less than 0.05 were considered as significant (Graphpad Prism 5.0). This experiment was performed in triplicate and the results were expressed as mean ± standard deviation (SD).

The emulsion used in penetration studies was an extemporaneous dispersion, but maintained its initial characteristics while these experiments were carried out. 

#### 2.3.6. Electrical Stability of Formulations and ZnPc

A volume of 1 mL of the formulations containing about 4.5 μg/mL of ZnPc was placed in beakers containing Ag/AgCl electrodes and subjected to a constant electric current of 0.5 mA for 1 h. The mean size of the emulsion and nanoemulsion and ZnPc concentration in each formulation were determined before and after the application of the electric current. 

### 2.4. Quantification of ZnPc

ZnPc was quantified using spectrofluorimetry (Shimadzu RF-1501, Kyoto, Japan), setting the excitation wavelength at 610 nm and monitoring the fluorescence emission in the range of 630–800 nm. Slits were adjusted to 5 for both excitation and emission settings. A linear calibration graph of ZnPc in DMSO was obtained over a working concentration range of 10–200 ng/mL (*y* = 34.03*x* + 4.325; *r* = 0.999). Intraday and inter-day precision and accuracy of the method showed a variation coefficient not greater than 4%. The limits of quantification and detection of the method were 3.8 and 1.14 ng/mL, respectively. 

To determine the concentration of ZnPc incorporated in the formulations, they were diluted in DMSO (1:80) and analyzed in the spectrofluorimeter using the parameters and the calibration graph described above. Blank formulations diluted in the same manner did not interfere in quantification.

To quantify the ZnPc in the skin, a matrix-matched calibration curve was constructed. For this, dermatomed (~700 μm thickness) pig ear skin of about 1.4 cm^2^ was punctured and spiked with known concentrations of ZnPc standard solutions in DMSO, each concentration in quintuplet. ZnPc was then extracted from the contaminated skin using 5 mL of DMSO. For this, skin samples were vortex-mixed (IKA Works Inc., Wilmington, NC, USA) for 2 min, homogenized using a tissue homogenizer (T25 Digital, IKA Works Inc., Wilmington, NC, USA) for 1 min at 13,500 rpm and bath-sonicated (Quimis Q335D model, 40 kHz, São Paulo, Brazil) for 30 min. The resulting dispersion was centrifuged at 20,000× *g* for 10 min, the resulting supernatant was filtered through a 0.45 μm polytetrafluoroethylene (PTFE) membrane, and the ZnPc amount was fluorometrically assayed in the filtrate using the parameters described above. A linear calibration curve (*y* = 27.15*x* + 756.4; *r* = 0.991) was obtained over a working concentration range of 10–200 ng/mL. Samples resulting from the in vitro skin penetration studies were analyzed and quantified using this calibration curve.

### 2.5. In Vitro Passive Skin Penetration Studies

Skin from the outer surface of a freshly excised porcine ear, obtained from a local slaughterhouse (Ipuã, São Paulo, Brazil), was carefully dissected, dermatomed (~700 μm thickness), stored at −20 °C and used within one month. Before the experiments, the skin was mounted in a Franz-type diffusion cell (diffusion area of 0.95 cm^2^), with the stratum corneum facing the donor compartment and the dermis facing the receptor medium. An infinite dose (i.e., the donor concentration is constant and significantly greater than the receptor) of ZnPc formulations at about 4.5 µg/mL of ZnPc was applied to the donor compartment (n = 4 for each). The receptor compartment (15 mL) was filled with isotonic PBS (10 mM, pH 7.4, 0.9% NaCl) containing sodium dodecyl sulfate (SDS) at 1% to guarantee sink conditions. The solubility of ZnPc in the receptor medium (PBS with SDS 1%) was 12.5 ± 0.1 µg/mL. Throughout the experiment, the receptor medium was constantly stirred. 

After 30 or 60 min of contact of the formulations with the skin, the skin surfaces were carefully rinsed with distilled water to remove any excess formulation and carefully wiped with tissue paper. The stratum corneum was separated from viable epidermis and dermis ([E + D]) using a validated tape stripping technique [24] with 15 pieces of adhesive tape (Scotch shipping packaging tape 3 M, São Paulo, Brazil). The surface of the skin was first thoroughly cleaned with water and subsequently with a dry paper towel to remove any excess formulation. The drug was extracted from the 15 strips collectively before the quantitative analysis; the tape strips containing the stratum corneum were immersed in 5 mL of DMSO, vortex-stirred for 2 min and sonicated for 30 min in an ultrasonic bath. The DMSO phase was filtered through a 0.45 μm PTFE membrane, and the resulting filtrate was fluorometrically assayed for ZnPc concentration. The remaining skin [E + D] was cut into small pieces, vortex-mixed for 2 min in 3 mL of DMSO, homogenized by a tissue homogenizer for 1 min at 13,500 rpm and bath-sonicated for 30 min. The resulting mixture was centrifuged at 20,000× *g* for 10 min and the supernatant was filtered through a 0.45 μm membrane, and the ZnPc amount in the filtrate was fluorometrically assayed. 

Before and at the end of the experiments, both the donor and receiver compartments were filled with PBS to determine the electrical skin resistivity (R) and guarantee the integrity of the stratum corneum for the experiments [28]. Briefly, an alternating current generated at 100 mV and 10 Hz (Arbitrary Waveform Generator 33220A model, 20 MHz function, Agilent, Santa Clara, CA, USA) was applied through the skin mounted in the diffusion cell. The skin electrical current was measured using a multimeter and the skin electrical resistance was calculated using Ohm’s law. Skin samples with R under 35 kΩ cm^2^ were considered damaged and were not used in the experiments.

### 2.6. In Vitro Iontophoresis

The skin was mounted on the diffusion cell and its initial R was determined as previously described. Infinite doses of ZnPc nanoemulsion or emulsion at about 4.5 µg/mL of ZnPc (n = 4 for each) were then put in contact with the skin in the donor compartment. The receptor chamber was filled with 15 mL of isotonic PBS pH 7.4 under 500 rpm stirring. Anodal iontophoresis was performed using Ag/AgCl electrodes, with the Ag (anode) and AgCl (cathode) electrodes inserted into the donor and receptor compartments, respectively. A constant electric current at a density of 0.5 mA/cm^2^ was applied for 30 or 60 min using a power supply (Digital SourceMeter2400, Keithley, Beaverton, OR, USA). At the end of the experiments, the stratum corneum was separated from the rest of the skin using the tape stripping technique, and the ZnPc extracted from the stratum corneum and from the [E + D] (as described previously) was quantified by spectrofluorimetry.

The number of ions required for the passage of the 0.5 mA/cm^2^ electric current for the maximum time of 60 min of the experiment, was calculated according to Equation (2):

T = (i × t)/F,(2)

where “T” is the number of moles required to carry the electric current, “i” is the electric current applied expressed in ampere, “t” is the electric current application time in seconds and “F” is the Faraday constant (96,500 C).

### 2.7. In Vivo ZnPc Penetration in Tumor

#### 2.7.1. Animals and Tumor Induction

Female C57BL6 mice, 6–8 weeks (20 g average body mass) were purchased from the animal facility center of the University of São Paulo, Campus of Ribeirão Preto. All animal experimental protocols and procedures were approved on 4 May 2018 by the University of São Paulo Animal Care and Use Committee (Authorization number: 17.5.344.60.00) which is in accordance with the National Institutes of Health Guidelines for the Care and Use of Laboratory Animals. The animals were kept in clean cages (7020 cm^2^, 6 animals per cage), exposed to daily 12:12 h light-dark cycles and natural moisture, under local temperature control, maintained between 20 and 24 °C, natural humidity. 

One day before tumor induction, the animal’s back hairs were removed by trichotomy. Murine melanoma cells (B16F10) were obtained from the American Type Culture Collection (Rockville, MD, USA). The cells were cultured in Dulbecco’s Modified Eagle’s Medium (DMEM, pH 7.2–7.4) (Gibco, Grand Island, NY, USA) containing 10% (*v*/*v*) of heat-inactivated fetal bovine serum (Gibco, Grand Island, NY, USA), 10,000 U/mL of G penicillin and 10,000 g/mL of ciprofloxacin at 37 °C with 5% CO_2_ and a humidified atmosphere. The tumor was induced by subcutaneous injection of 10^6^ cells of B16F10 in the dorsal region of the mice. Treatments started 10 days after tumor induction, when the tumors reached a volume of about 100 ± 50 mm^3^.

#### 2.7.2. Treatment

To evaluate the in vivo ZnPc penetration into the tumor, the animals were divided into three groups of two animals each and subjected to topical treatment with the ZnPc nanoemulsion using three different times of application of anodic iontophoresis (0.5 mA/cm^2^): 5, 15 and 30 min, with one time period being allocated to each discrete group of animals. Before the treatment, the animals were anesthetized by intraperitoneal injection of a mixture of 30% ketamine and xylazine (1:2) in saline solution at a dose of 100 μL/animal. 

**Figure 1:**
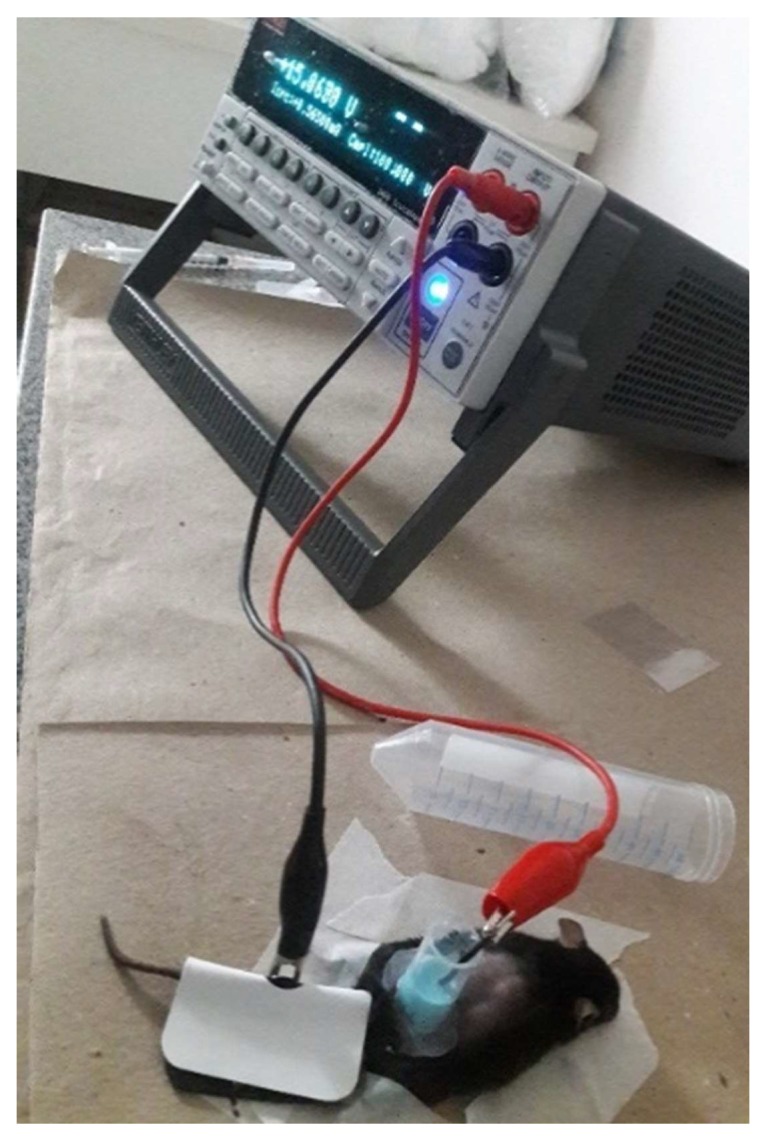
Photograph of the in vivo topical treatment with nanoemulsion iontophoresis application.

ZnPc nanoemulsion was applied over the tumor using an open plastic chamber that was fixed to the skin using silicone grease (Figure 1). Anodal iontophoresis was applied at 0.5 mA/cm^2^ by placing the positive electrode in contact with the formulation and fixing a negative patch (Iomed, Salt Lake City, UT, USA) on the tail of the mouse as a counter electrode [29].

The animals were sacrificed after the treatments and the tumor was immediately removed for analysis.

#### 2.7.3. Confocal Scanning Laser Microscopy Analysis

The tumor was separated from the skin to recover tumor mass, and both skin and tumor had their fluorescence preserved by soaking them in Tissue-Tek^®^ (O.C.T. Compound) before being frozen at −80 °C. Cryosections (20 µm) perpendicular to the skin surface were made using a cryostat (Leica CM1860, Buffalo Grove, IL, USA). All slices received Fluoromount to avoid photobleaching during analysis. For confocal fluorescence microscopy, a Leica TCS SP8 confocal microscope (Mannheim, Germany) with a 20× immersion objective was used. Samples were excited with a laser at 638 nm and the fluorescence was detected at 640–800 nm. Untreated skin and tumor samples were used to adjust the parameters of the equipment so that the autofluorescence of the tissues did not interfere in the analyses.

### 2.8. Statistical Analysis

The statistical analyses of stability and in vitro skin penetration studies were performed using one-way ANOVA, with Tukey’s post hoc test (*p* < 0.05 was considered the minimum value of significance). The other results were analyzed by the student *t*-test (*p* < 0.05 was considered the minimum value of significance).

## 3. Results

### 3.1. Emulsion and Nanoemulsion Characterization

Table 1 shows the physicochemical characteristics of the emulsion and nanoemulsion prepared with an excess of ZnPc, together with the concentration of drug solubilized in each formulation.

It can be seen from Table 1 that the nanoemulsions prepared with ultrasound aid, had droplet sizes of the order of nanometers, 70 times smaller than that of the emulsion droplets. The nanoemulsion droplet size shows a narrow distribution (small PDI), while the size variation of the emulsion droplets was broad (high SPAN). The zeta potential of the ZnPc emulsion was significantly higher than that of the ZnPc and blank nanoemulsion (ANOVA, with Tukey’s post hoc test, *p* < 0.05); however, all the formulations were cationic. Addition of the drug to the blank nanoemulsion did not significantly alter the mean droplet size and distribution. The pH of ZnPc emulsion and ZnPc nanoemulsion was similar (*p* > 0.05), near to 6.5. 

**Figure 2:**
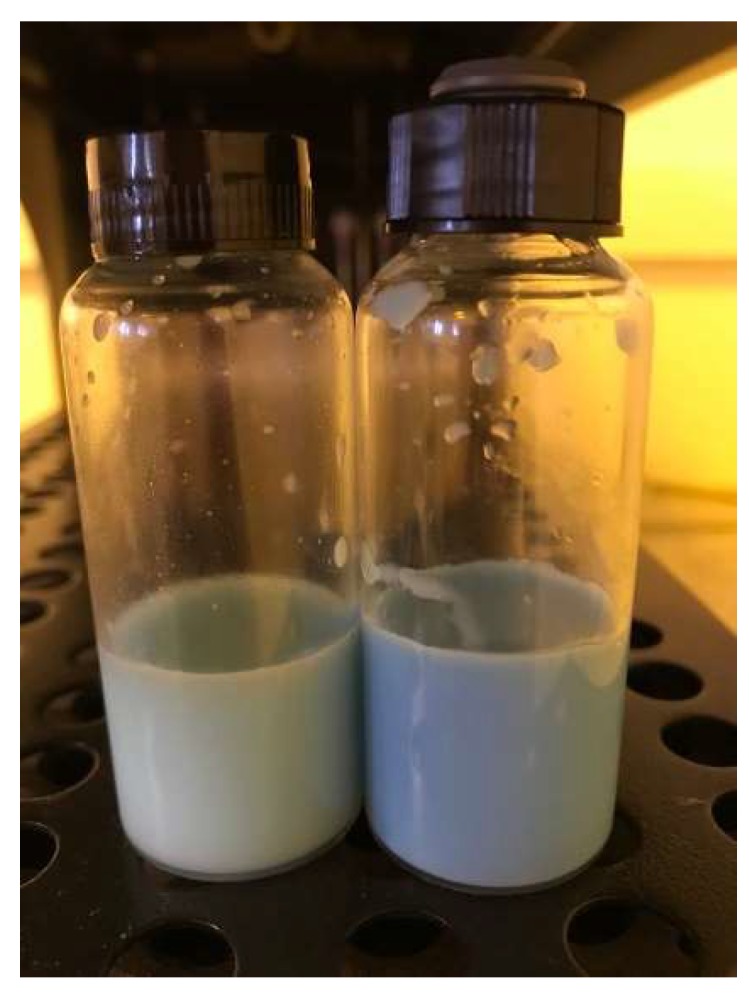
Macroscopic aspect of the emulsion (**left**) and nanoemulsion (**right**) containing ZnPc (picture taken in a yellow light environment to avoid photodegradation of ZnPc).

Despite the larger size of the emulsion droplets, it solubilized 2.5 times less ZnPc than the nanoemulsion. Figure 2 clearly shows the color differences between the formulations due to the different concentrations of solubilized ZnPc, which has a blue color. 

The size and zeta potential of formulations prepared with similar concentrations of ZnPc and PBS as dispersant phase can be seen in Table 2.

Preparation of the formulations using PBS in place of water did not significantly alter the mean size and distribution of particles, with nanoemulsion continuing to exhibit particle sizes about 70-times smaller than those presented by the emulsion. The zeta potential, in turn, decreased, but the dispersions remained cationic, with no significant difference between emulsion and nanoemulsion (*t*-test, *p* < 0.05).

Formulations prepared with PBS have 0.9% NaCl, i.e., 154 μmol/mL. According to Equation (2) for the iontophoresis experiments, approximately 20 μmols of NaCl are required to carry the electric current of 0.5 mA for 1 h. The formulations therefore have 8 times more ions than required, ensuring the passage of the electric current during the iontophoretic experiments.

The exact amount of ZnPc incorporated into the emulsion and the nanoemulsion was 4.56±0.05 and 4.42 ± 0.49 µg/mL, respectively. 

**Figure 3:**
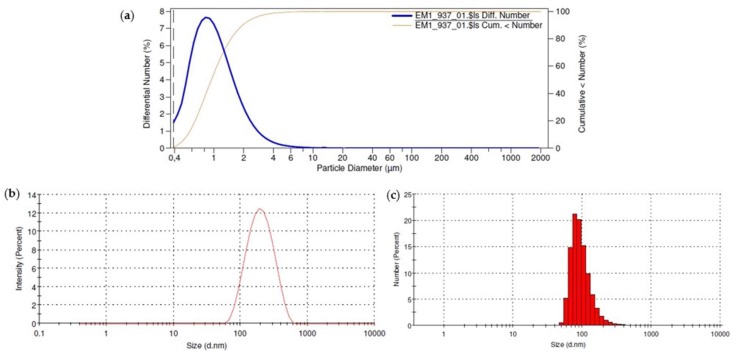
Size distribution of (**a**) ZnPc emulsion (**b**) ZnPc nanoemulsion by intensity and (**c**) ZnPc nanoemulsion by number.

Figure 3 shows the size distribution of the formulations in PBS. It can be seen that both emulsion and nanoemulsion presented a single population of droplets, i.e., a monomodal droplet size distribution. For the emulsion, 90% of the droplets were smaller than 18 μm, whereas for nanoemulsion 87% of the nanodroplets were between 50 and 120 nm.

**Figure 4:**
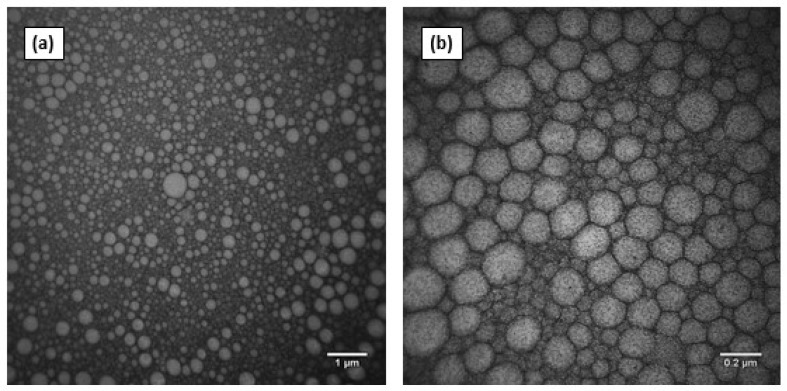
TEM of ZnPc nanoemulsion stained with negative contrast of 2% uranyl acetate obtained with magnification of (**a**) 20,000×; (**b**) 100,000×.

TEM images of ZnPc nanoemulsion (Figure 4) confirmed the narrow distribution of the nano-size droplets, mostly less than 200 nm, corroborating the results of DLS analysis (Table 2), and showed the spherical and regular shape of the droplets.

### 3.2. Stability Study

**Figure 5:**
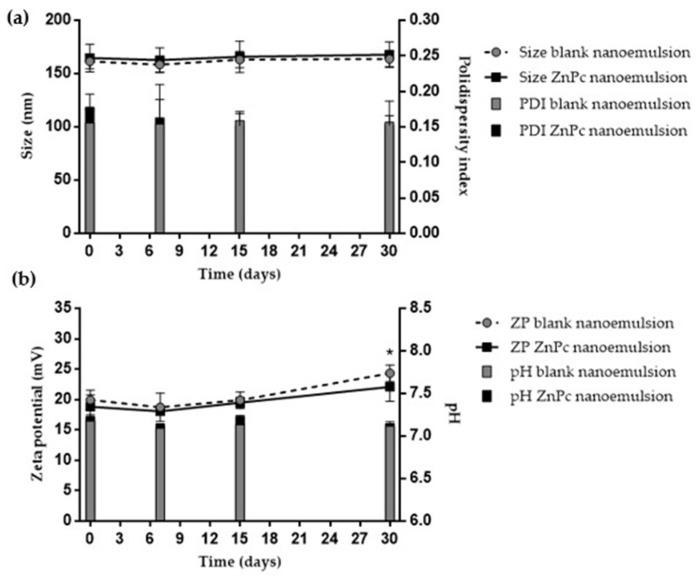
Physical stability of the blank and ZnPc nanoemulsion during 60 days of storage at 4 °C for: (**a**) Size and polydispersity index (PDI), (**b**) Zeta potential and pH. Statistical analysis was performed by size, PDI and zeta potential * indicates statistical difference in relation to time 0 (ANOVA with Tukey’s post hoc test, *p* < 0.05).

Figure 5 shows the characteristics of size, PDI, zeta potential and pH of the blank and ZnPc nanoemulsions in PBS as a function of time, stored at 4 °C.

No variation of size, PDI, zeta potential and pH of the ZnPc nanoemulsions was observed during the 30 days of the experiment, as can be observed in Figure 5. On the other hand, emulsions prepared with PBS, although apparently stable in the absence of ions (formulation made using water as dispersion medium instead of PBS), showed phase separation after 10 h of preparation. 

Table 3 shows the stability of emulsion and nanoemulsion freshly prepared in PBS, both containing the same concentration of ZnPc, in the presence of the electric current (0.5 mA for 1 h). 

It is possible to notice in Table 3 that the application of the constant electric current to the formulations did not significantly change their size (*t*-test, *p* < 0.05). ZnPc concentration also remained constant (*t*-test, *p* < 0.05). Therefore, for the in vitro penetration studies, emulsion and nanoemulsion containing about 4.5 μg/mL of ZnPc were prepared using PBS as the dispersant phase and used just after the preparation.

### 3.3. In Vitro Skin Penetration Studies

**Figure 6:**
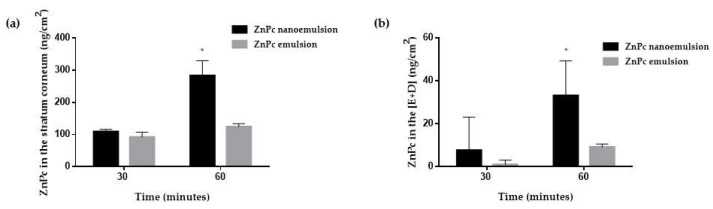
In vitro passive experiments: ZnPc recovered from (**a**) stratum corneum and (**b**) viable skin (E + D) after passive treatment of skin with ZnPc nanoemulsion (black columns) and emulsion (gray columns). * significant statistical difference (ANOVA with Tukey’s post hoc test, *p* < 0.05).

Figure 6 shows the amount of ZnPc recovered from the stratum corneum and [E + D] after 30 and 60 min of passive penetration experiments with ZnPc nanoemulsion and ZnPc emulsion. 

Passively, after 30 min, skin treatment with the emulsion or nanoemulsion resulted in similar amounts of ZnPc in the skin. After 60 min however, treatment with nanoemulsion showed 2.3 and 3.6-fold more ZnPc in the stratum corneum and [E + D] respectively, than treatment with emulsion. 

**Figure 7:**
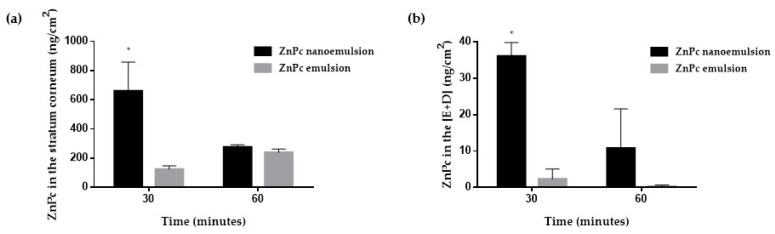
Iontophoresis in vitro: ZnPc recovered from (**a**) stratum corneum and (**b**) viable skin (E + D) after iontophoretic treatment of skin with ZnPc nanoemulsion (black columns) and emulsion (gray columns). * significant statistical difference (ANOVA with Tukey’s post hoc test, *p* < 0.05).

The influence of iontophoresis on the penetration of ZnPc as a function of treatment time and formulation is shown in Figure 7. 

Iontophoretic treatment with the nanoemulsion resulted in an increase of about 5-fold of ZnPc in the stratum corneum relative to the emulsion treatment, in the first 30 min (Figure 7a). This increase was even more significant at [E + D] (Figure 7b), whereby almost 16-fold more ZnPc was recovered after iontophoresis of the nanoemulsion than after iontophoresis of the emulsion. 

After 60 min of iontophoresis of the emulsion, the amount of ZnPc in the stratum corneum increased compared to the 30 min of treatment (Figure 7a, gray bars, *t*-test, *p* < 0.05). This increase was not reflected however in [E + D], and the amount of ZnPc found there after 60 min of iontophoresis was similar to that recovered after 30 min (Figure 7b, gray bars). On the other hand, the iontophoresis of the nanoemulsion for 60 min resulted in a decrease in the amount of ZnPc in both the stratum corneum (Figure 7a, black bars) and [E + D] (Figure 7b, black bars) in relation to the 30 min treatment. 

The concentration of ZnPc in the receptor solution was below the limit of quantification of the analytical method. 

Table 4 shows the ratio of penetrated ZnPc after iontophoresis and passive treatment for each dosage form at each treatment time.

In comparison to passive penetration, iontophoresis clearly increased ZnPc in the skin after 30 min of treatment, regardless of the dosage form, emulsion or nanoemulsion, but mainly for nanoemulsion treatment (6-fold in the stratum corneum and almost 5-fold in [E + D]) (Table 4). After 60 min however, iontophoresis does not appear to have influenced or even decreased, especially for nanoemulsion, the amount of ZnPc in the skin relative to the passive treatment (Table 4). As the application of the electric current does not degrade the ZnPc (Table 3), this decrease in its concentration in the skin when iontophoresis is applied for a longer time may be related to a greater permeation of the drug through the skin with iontophoresis. However, the sensitivity of the analytical method was not sufficient for it to be detected in the receptor compartment of the in vitro penetration study.

### 3.4. In Vivo ZnPc Penetration in Skin Tumor

**Figure 8:**
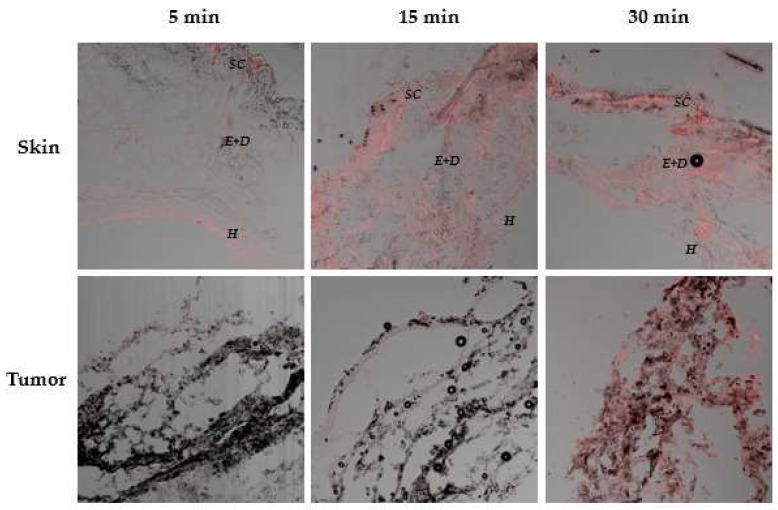
Representative confocal images of 20 μm thickness cryo-sections perpendicular to the skin surface and tumor samples after different times of ZnPc nanoemulsion iontophoresis (SC: stratum corneum, E + D: Epidermis and Dermis, H: hypoderm). The images represent the overlap of the light and dark field. ZnPc fluorescence was observed in the red channel (εexc = 638, εem = 640–800 nm) 20× magnification.

Figure 8 shows red fluorescence, a characteristic of ZnPc detected in skin and tumor samples after 5, 15 and 30 min of ZnPc nanoemulsion iontophoresis in animal-induced tumors. 

The fluorescence of ZnPc can be observed in the skin that covers the tumor from the first minutes of iontophoresis. In order to be visualized in the tumor however, it took 30 min of iontophoresis. This period of application was sufficient for the ZnPc to distribute homogeneously throughout the tumor.

## 4. Discussion

The success of PDT depends, within other factors, on the PS penetrating the skin and reaching cancer cells that should be eliminated. Nanoemulsification of hydrophobic PS is an alternative to the application and efficacy of these drugs in PDT since the low solubility precludes its administration. In this work, we developed and characterized an emulsion and a nanoemulsion with exactly the same composition for the delivery of ZnPc, a second generation PS, and evaluated the influence of droplet size and iontophoresis on this drug skin penetration. 

ZnPc nanoemulsion was efficiently obtained using ultrasound. Both emulsion and nanoemulsion dispersions showed a positive zeta potential value due to the presence of the cationic lipid DOTAP in their composition. Dispersions with high zeta potential have their stability increased due to repulsion between the particles that prevent their aggregation [30]. Moreover, the positive zeta potential of the dispersions may contribute to a better interaction of the formulations with the skin and to a greater efficiency of the anodic iontophoresis. This is because due to the low pKa of the carboxylate groups of the fatty acids that compose the stratum corneum [31], skin presents net negative charges when in contact with formulations at physiological pH [32], such as those developed in this work (Table 2). In this way, cationic particles interact better with the skin than anionic particles [33,34]. Furthermore, negative charges of the stratum corneum favor the electrosmotic flow from the anode to the cathode when iontophoresis is applied [19] and the positive zeta potential of the dispersions should favor electromigration [35], contributing to the electric transport of the drug through the skin by anodic iontophoresis.

Emulsions and nanoemulsions were prepared with an identical composition to first assess their ability to solubilize the highly lipophilic ZnPc. Nanoemulsions, as seen in Table 1, were able to solubilize a quantity of ZnPc 2.5 times higher than the emulsion and also showed a narrow droplet size distribution (Table 1). These properties could also allow for the application of this nanoemulsion by other routes, such as intravenously. 

It is noteworthy that the developed nanoemulsion was able to solubilize a higher concentration of ZnPc than other lipid release systems described in the literature [36,37,38,39]. For instance, ZnPc was incorporated in liposome [36], microemulsion [37], and nanoemulsion [38] at the concentrations of 1 µg/mL, 7 µg/mL and 35 µg/mL, respectively, while the nanoemulsion developed in this work incorporated about 50 µg/mL of ZnPc (Table 1).

Several studies have demonstrated the ability of nanostructured systems to increase the penetration of PS [40,41,42,43,44,45]. However, none of them have used as a control a formulation containing the same components in the same concentrations, but with large particle size, in order to evaluate exclusively the effect of the reduced size of the particles in PS skin penetration. In order to allow for the application of iontophoresis and to guarantee adequate transport of the electric current (Equation (2)), it was decided to prepare the formulations in isotonic PBS instead of ultrapure water. This modification changed the pH from 6.4 to about 7, since PBS was used as dispersant, and slightly decreased the zeta potential of the dispersions (Table 2), although they remained cationic, allowing for the application of anodic iontophoresis. The other characteristics, such as size and polydispersity (Table 2 and Figure 3) and morphology (Figure 4), were similar to those obtained for formulations in the absence of ions, i.e., in water (Table 1). 

The stability of the ZnPc nanoemulsions obtained in PBS was adequate, maintaining the parameters of size, PDI, zeta potential and pH for up to 30 days (Figure 5). However, the addition of the ions necessary for buffer preparation altered the equilibrium of the emulsion, and it separated after a short period of time. Thus, to study the influence of droplet size on skin penetration of ZnPc, formulations were always prepared immediately prior to the skin and tumor penetration studies. The stability of both in function of the electric current application time, 60 min, was also verified. As can be observed in Table 3, there was no significant change in size or concentration of ZnPc in the presence of the electric current of 0.5 mA for 60 min, allowing for the accomplishment of the skin penetration experiments with both emulsion and nanoemulsion. 

In vitro passive skin penetration studies showed that the reduction of droplet size favored ZnPc penetration into the skin (Figure 6), since higher concentrations of drug were quantified in the stratum corneum and in the [E + D] when nanoemulsion was applied. The dynamic characteristics of dispersions of immiscible liquids stabilized by emulsifying agents allied with the small size of the nanoemulsion droplets, favors the interactions with skin cells and may cause certain disorder of the stratum corneum, consequently facilitating drug penetration [27].

It was possible to verify that although the concentration of penetrated ZnPc when incorporated into the nanoemulsion was greater than that of emulsion, most of the ZnPc was quantified in the stratum corneum and a small amount reached the [E + D]. It is known that lipophilic drugs such as ZnPc, present a great affinity for the stratum corneum [46], being retained there rather than partitioning to the layers of skin where the tumor cells settle. In addition, a tumor mass itself, along with its peculiar microenvironment, provides a significant barrier to the transport of drugs.

An iontophoresis physical enhancement method can be used together with nanocarriers to induce a synergistic penetration enhancement of drugs into/through the skin [29,35,47,48]. Thus, in vitro iontophoresis penetration was performed with ZnPc emulsion and nanoemulsion to increase penetration of ZnPc and also to investigate whether such an increase occurs in the same proportion regardless of the dispersion droplet size in which the drug is delivered.

The results revealed that both emulsion and nanoemulsion subjected to 30 min of iontophoresis increased the penetration of ZnPc into the skin compared to passive delivery (Figure 7 and Table 4). Nanoemulsion iontophoresis was more effective, resulting in higher amounts of ZnPc in both the stratum corneum and [E + D]. Longer iontophoresis times (60 min) doubled the ZnPc penetration in the stratum corneum when emulsion was applied, but decreased the amount of drug found in the [E + D] (Figure 7 and Table 4). For nanoemulsion, the 60 min iontophoresis resulted in a reduction of more than 2-fold over 30 min, of ZnPc in the entire dermatomed skin. 

It is assumed that the drug concentration decreased in the skin with a longer iontophoresis time by reaching the receptor solution. Although the experiment was conducted using an infinite source of drug, it was only performed for 1 h. Therefore, it is plausible to assume that steady state was not achieved and the amount of drug arriving at the receptor solution was still less, after 1 h, than the amount found in the skin layers. The amount of drug arriving in the receptor solution was, however, diluted in 15 mL of the receptor medium, unlike that found in the skin, which was extracted with a maximum of 5 mL of DMSO. As the limit of quantification of the analytical method was about 4 ng/mL, it is reasonable to assume that the permeated drug was not quantified in the receptor medium due to sensitivity of the analytical method. Therefore, as the drug does not degrade in the presence of the electric current (Table 3) and the amount of drug that permeates the skin as a function of time is cumulative, it is possible to suggest that iontophoresis enhanced skin permeation of ZnPc, which reached the receptor solution, but cannot be quantified therein due to the sensitivity of the analytical method. In other words, it is believed that iontophoresis pushes the drug into the receptor medium, resulting in less drug quantification in the skin layers at least while steady state is not achieved. 

It is interesting to note that the nanometric droplets of the nanoemulsion compared to the emulsion also seem to facilitate the transport of electrical current through the skin, since more ZnPc crosses the skin when nanoemulsion was subjected to iontophoresis.

The fact that ZnPc seems to be able to cross the skin and reach the receptor medium with iontophoresis is advantageous because it confirms that electric current application may be able to overcome the tumor barrier when it is present and allow the drug to reach the tumor cells. 

To verify whether iontophoresis of the nanoemulsion is capable of enhancing the penetration of ZnPc into the tumor, in vivo studies in a melanoma murine model were performed. Iontophoresis was applied in vivo for up to 30 min because this was the time when the highest amount of ZnPc was observed in [E + D] in in vitro iontophoresis (Figure 7). 

Iontophoresis of the ZnPc nanoemulsion directly into the tumor site allowed the penetration of ZnPc into the skin shortly after 5 min of application, although the fluorescence intensity of the drug increased with increasing iontophoresis time (Figure 8). After the first 5 min of iontophoresis, ZnPc penetration appears to occur primarily through cutaneous appendices and furrows, with accumulation of the lipophilic drug in the hypodermic layer. Over time, however, the characteristic red fluorescence of ZnPc can be seen throughout the skin, probably due to a better distribution of the nanoemulsion in the skin. Only after 30 min of iontophoresis it was possible to visualize the ZnPc in the tumor. Therefore, a longer electric current application time is necessary to favor the entrance of a greater concentration of ZnPc able to overcome the tumor barrier and reach the tumor cells. In addition, the presence of ZnPc in the tumor after 30 min of iontophoresis confirmed the hypothesis that the smaller amount of ZnPc found in the skin after 60 min of in vitro nanoemulsion iontophoresis (Figure 7) resulted from increased permeation of the drug through the skin reaching the receptor solution. 

It is interesting to note that the tumor used in the studies was of the melanoma type. One of the major obstacles in the treatment of melanoma by PDT is the presence of chromophores such as melanin, hemoglobin, lipids and proteins in the skin and melanoma tumor that compete with the PS for light employed during PDT [49]. Consequently, high concentrations of ZnPc in the tumor are required for the PDT response to be effective even if it is a second generation PS that absorbs light in lengths more suitable for the PDT application. Visualization of the fluorescence relative to ZnPc in the tumor (Figure 8) is a powerful indicator that the PS that was able to distribute in the tumor, is photoactive.

It is known that in vitro, about 8–15 ng/mL (15–25 nM) of ZnPc undergo cellular uptake by tumor cells of different lines and are able to induce tumor cell death when exposed to PDT [50,51]. It can be inferred that the amount of ZnPc that reaches the viable epidermis after 30 min of iontophoresis (Figure 7b), of approximately 36 ± 4 ng/cm², is promising to reach the tumor, as shown in in vivo studies (Figure 8), and induce tumor death in the presence of light. However, PDT experiments are certainly needed to prove this.

The direct application of the PS in the tumor site as proposed in this work, instead of administration of a pro-PS, such as 5-ALA, has advantages over the protocol currently used in topical PDT. The FDA approved topical PDT protocol [52] involves the use of a solution of 5-ALA at 20% (LEVULAN^®^) that must be applied over the tumor lesions at the doctor´s office or hospital, 14 to 18 h before irradiation by light; after this time the patient must return again to perform the irradiation. With the administration of ZnPc nanoemulsion by iontophoresis, it would take only 30 min to apply the formulation, with the patient already in the hospital, followed by PDT.

## 5. Conclusions

In summary, the smaller the droplet size of the emulsion, the greater the skin penetration of the lipophilic drug solubilized therein. Iontophoresis of the nanoemulsion is a good approach to carry the highly lipophilic and photoactive ZnPc through the skin up to the tumor. Certainly, further studies are needed to verify the effect of PDT on tumors after iontophoresis of the ZnPc loaded nanoemulsion. The influence of nanoemulsion on uptake of the drug by tumor cells also still needs to be studied. In general, this work suggests that nanoemulsions can be used as a platform for the iontophoresis of other lipophilic drugs of dermatological interest, such as antifungal, anti-inflammatory and antihistaminic drugs, which need to penetrate in high concentrations in deep layers of the skin, in a short period of application, to be more effective.

## Figures and Tables

**Table 1:** Physicochemical characterization of the formulations and concentration of solubilized ZnPc.

Characteristics	Formulation		
	ZnPc Emulsion	Blank Nanoemulsion	ZnPc Nanoemulsion
Size (nm) ^a^	12.8 × 10^3^ ± 3.5 × 10^3^ *	177 ± 16	173 ± 2
PDI or SPAN ^b^	2.04 ± 0.30 *	0.17 ± 0.01	0.20 ± 0.02
Zeta potential (mV)	63 ± 1 *	43 ± 2	43 ± 7
pH	6.4 ± 0.2	5.6 ± 0.1 *	6.4 ± 0.3
ZnPc concentration (μg/mL)	18.5 ± 7.4 *	-	49.4 ± 6.2

^a^ Mean size determined by laser diffraction and DLS (intensity) for emulsion and nanoemulsion, respectively. ^b^ PDI for size distribution by DLS and SPAN for size distribution evaluated by laser diffraction. Values expressed as mean ± SD of 3 replicates. * indicates statistical difference between values per line (ANOVA, with Tukey’s post hoc test, *p* < 0.05).

**Table 2:** Physicochemical characteristics of emulsion and nanoemulsion dispersed in PBS containing about 4.5 μg/mL of ZnPc.

Characteristics	Formulation	
	ZnPc Emulsion	ZnPc Nanoemulsion
Size (nm) ^a^	14.4 × 10^3^ ± 5.7 × 10^3^ *	176 ± 6
PDI or SPAN ^b^	2.4 ± 0.4 *	0.17 ± 0.02
Zeta potential (mV)	25 ± 4	24 ± 3
pH	7.19 ± 0.04	7.14 ± 0.02

^a^ Mean size determined by laser diffraction and DLS (intensity) for emulsion and nanoemulsion, respectively. ^b^ PDI for size distribution by DLS and SPAN for size distribution evaluated by laser diffraction. Values expressed as mean ± SD of 3 replicates. * indicates statistical difference between values per line (*t*-test, *p* < 0.05).

**Table 3:** Stability of the formulations in the presence of the electric current at 0.5 mA for 60 min.

Formulation	Size (nm) *		ZnPc Concentration (µg/mL)	
	Before Electric Current	After Electric Current	Before Electric Current	After Electric Current
ZnPc emulsion	14.4 × 10^3^ ± 5.7 × 10^3^	11.7 × 10^3^ ± 3.7 × 10^3^	4.43 ± 0.21	4.43 ± 0.15
ZnPc nanoemulsion	166 ± 3	158 ± 5	4.43 ± 0.15	4.40 ± 0.17

* Mean size determined by laser diffraction and DLS (intensity) for emulsion and nanoemulsion, respectively. Size and ZnPc concentration expressed as mean ± SD of 3 determinations.

**Table 4:** Ratio between ZnPc recovered from the skin after iontophoresis and passive treatment.

Formulation	ZnPc Iontophoresis/Passive Penetration			
	Stratum Corneum		Viable Skin [E + D]	
	30 min	60 min	30 min	60 min
Emulsion	1.3	1.9	2.3	0.03
Nanoemulsion	6	0.9	4.7	0.33
